# The Works of Alexander Monro, M.D. F.R.S. Fellow of the Royal College of Physicians, and late Professor of Medicine and Anatomy in the University of Edinburgh.

**Published:** 1781-05

**Authors:** 


### THE
### LONDON MEDICAL JOURNAL,
### For MAY 1781.

### SECTION I.
### BOOKS.

I. *The Works of* Alexander Monro, M. D. F. R. S. *Fellow of the Royal College of Physicians, and late Professor of Medicine and Anatomy in the University of Edinburgh. Published by his Son,* Alexander Monro, M. D. *President of the Royal College of Physicians, and Professor of Medicine and of Anatomy and Surgery in the University of Edinburgh. To which is prefixed, the Life of the Author.* 4to. Edinburgh 1781, 791 pages, with eight * copper plates.

THE late Dr. Monro, besides his anatomy of the bones and nerves, was the author of a great number of other ingenious productions, most of which were ori-

* One of these is an engraving, executed by Mr. Basire, from an excellent portrait of the author, by Allan Ramsay, Esq.

ginally publiſhed in the Edinburgh medical Eſſays. It gives us pleaſure to ſee all the works of this juſtly celebrated writer, brought together in a collection like the preſent. To thoſe printed formerly under his own inſpection, the editor has added two pieces. One of theſe is an oration *de Cuticula Humana*, delivered by him above forty years ago in the Univerſity of Edinburgh, in which many curious circumſtances are deſcribed that had eſcaped the obſervation of former anatomiſts, particularly the appearance of the fibres that connect the *cuticula* to the *cutis vera*; the other piece is *An Eſſay on comparative Anatomy*, compoſed from notes taken at his lectures and publiſhed in 1744, but without his knowledge. In the preſent edition, the errors that had crept into the ſurreptitious impreſſion of that eſſay are corrected, and ſome few additions made to it from obſervations collected by the author with a view to a larger work on the ſubject, but which, by various avocations, he was prevented from proſecuting.

Beſides the pieces here publiſhed, it ſeems that he left ſeveral manuſcripts on different anatomical and practical ſubjects, which, from their not being inſerted, were probably not deemed

ſufficiently

ſufficiently intereſting or perfect for the public eye. Of theſe the principal are, *A Hiſtory of anatomical Writers—An Encheireſis Anatomica—Heads of many of his Lectures—A Treatiſe on Wounds and Tumours—Obſervations on ſome Parts of Heiſter's Surgery.*

For the gratification of our readers, we ſhall extract the following particulars from the account of the author written by his ſon Dr. Donald Monro, and prefixed to the preſent collection.

His father, who was the youngeſt of two ſons of Sir Alexander Monro of Bearcrofts, was bred to phyſic and ſurgery, and ſerved for ſome time as a ſurgeon in the army under King William in Flanders. He married Miſs Forbes, niece to Mr. Forbes of Culloden, and for ſome years ſucceſſively obtained leave of abſence from the army in winter, and reſided with his wife in London, where his ſon Alexander was born on the 8th of September, O. S. 1697. About three years after that period he quitted the army, and went to ſettle as a ſurgeon at Edinburgh, where he ſoon came into extenſive practice.

The ſon ſhewed an early inclination to the ſtudy of phyſic; and his father, after giving him the beſt education that Edinburgh then afforded, afforded, ſent him ſucceſſively to London, Paris, and Leyden, to improve himſelf further in his profeſſion.

At London he attended the lectures of Meſſ. Hawkſbee and Whiſton on experimental philoſophy, and the anatomical demonſtrations of Mr. Cheſelden. He at the ſame time employed himſelf much in diſſection; and having been admitted a member of a ſociety of young phyſicians and ſurgeons, who by rotation delivered diſcourſes on the uſes of the different organs, he read to them the firſt ſketch of his general account of the bones, which he afterwards publiſhed.

He ſent a great many anatomical preparations to his father, who depoſited them in the cabinet of curioſities which was then kept at Surgeons Hall in Edinburgh, and Mr. Adam Drummond, who was nominal profeſſor of anatomy to the Surgeons Company, was ſo well pleaſed with thoſe ſpecimens of his abilities, that he promiſed to reſign in his favour as ſoon as he ſhould return to Edinburgh.

From London he went to Paris, and from thence in 1718 to Leyden, where he ſtudied under the great Boerhaave, who ſoon conceived a high a high opinion of his talents, and wrote very favourably of him to his friends.

On his return to Edinburgh in 1719, he was appointed profeffor of anatomy to the Surgeons Company. Soon after this, his father prevailed on him to read fome public lectures on anatomy, and to illuftrate them by fhewing the preparations he had fent home from abroad; and without his knowledge, invited the Prefident and Fellows of the College of Phyficians, and the whole Company of Surgeons, to honour the firft day's lecture with their prefence. This unexpected company threw him into fuch confufion, as to make him entirely forget the words of the difcourfe he had written and committed to memory. Having left his papers at home, he was at a lofs for fome little time what to do; but having a ready prefence of mind, he immediately began to fhew fome of the anatomical preparations, in order to gain a little time for recollection; and very foon refolved not to repeat the difcourfe he had written, but to exprefs himfelf in fuch words as fhould occur to him from the fubject, which he was confident he underftood. The experiment fucceeded; he delivered himfelf well, and gained great applaufe as a good and ready fpeaker. After this, being perfuaded perſuaded that words expreſſive of his meaning would always occur in ſpeaking on a ſubject which he underſtood, he never during his whole life-time attempted, in teaching, to repeat the words of any written diſcourſe, but ſpoke from memory, and expreſſed himſelf eaſily, and even elegantly, in ſuch words as flowed from the ſubject.

In 1720, Dr. Alſton, then a young man, began at the requeſt of our author's father to give public lectures on botany; Dr. Monro undertaking at the ſame time to teach anatomy and ſurgery. Theſe were the firſt regular courſes of lectures on any of the branches of phyſic that had ever been read at Edinburgh, and may be conſidered as the foundation of that medical ſchool which has ſince acquired ſuch reputation all over Europe. Soon after that period, Profeſſorſhips of Anatomy and Phyſic were inſtituted in the Univerſity, and filled by our author, and by Drs. Sinclair, Rutherford, Innes, and Plummer; the Profeſſorſhip of Materia Medica and Botany, which Dr. Alſton then held, having been added to the Univerſity many years before.

The plan for a medical education at Edinburgh was ſtill incomplete without an hoſpital; a ſcheme a ſcheme was therefore propoſed by Mr. Monro, ſen. and others, for erecting one, and our author publiſhed a pamphlet, ſetting forth the advantages that would attend ſuch an inſtitution. In a ſhort time a ſmall houſe was fitted up for the purpoſe; and ſome years afterwards the Royal Infirmary was erected.

In order to make the hoſpital of ſtill further uſe to the ſtudents, our author frequently, while he continued profeſſor of anatomy, gave lectures on the ſurgical caſes; and in the year 1748, Dr. Rutherford began to deliver clinical lectures in the hoſpital.

From the time of his being received into the Univerſity, till the time of his reſigning in favour of his ſon the preſent profeſſor, a period of near forty years, he regularly every winter gave a courſe of lectures on anatomy and ſurgery, which laſted from October to May; and ſo great was the reputation he had acquired, that ſtudents flocked to him from the moſt diſtant parts of his Majeſty's dominions.

After he quitted the anatomical chair, he ſtill continued to read clinical lectures at the hoſpital, and to execute with the ſtricteſt punctuality the duties of ſeveral engagements both of a civil and political nature; for beſides being a Director

Director of the Bank of Scotland, he was a Juftice of the Peace, a Commiffioner of High Roads, &c.

He had the fatisfaction of affording to an aged father, every comfort that a man in the decline of life can well enjoy. The father, perfectly at eafe with regard to the neceffaries and conveniencies of life, faw with the utmoft pleafure an affectionate fon, efteemed and regarded by mankind, the principal actor in the execution of his favourite plan, the founding a feminary of medical education in his native country: and three years after the firft ftone of the new hofpital was laid, ended his days in a calm retreat at a pleafant country feat which his fon had purchafed in the county of Berwick.—The fon, who furvived him near thirty years, had the fatisfaction to behold this feminary of medical education frequented yearly by three or four hundred ftudents, and to fee it arrive at a degree of reputation exceeding his moft fanguine hopes.

In the beginning of the year 1725, our author married Mifs Ifabella Macdonald, fecond daughter of Sir Donald Macdonald of Macdonald in the Ifle of Sky, Baronet, by whom he had eight children, four of whom died young; the other four are ſtill living, viz. John Monro, Eſq; of Auchinbowie, Advocate, Counſellor at Law; Dr. Donald Monro, Phyſician in London; Dr. Alexander Monro, editor of the work before us; and Mrs. Philp, wife to James Philp, Eſq; Judge of the Court of Admiralty for Scotland. — To them our author proved a moſt affectionate father. In their youth he not only ſuperintended their education, but was himſelf their maſter in ſeveral branches; and when they grew up, he made them his companions and friends.

Dr. Monro was a man of a ſtrong muſcular make, of a middle ſtature, and poſſeſſed of great ſtrength and activity of body; but ſubject for many years to a ſpitting of blood on catching the leaſt cold, and through his whole life to frequent inflammatory fevers; which he uſed to attribute to the too great care his parents took of him in his youth, and to their having had him regularly blooded twice a year, which in thoſe days was looked upon as a great preſervative of health.

In the year 1762, he was attacked with the epidemical catarrhal fever called *influenza*, attended with pain and uneaſineſs in making water and going to ſtool, which remained ever after during his life, and were the firft fymptoms of that long and painful diforder, a fungous eroding ulcer of the rectum and bladder, which put an end to his life. The diforder increafed by flow degrees, and from May 1766 to July 10th 1767, the day on which he died, gave him fo conftant and great pain, that he had little refpite from it except by the power of opium.

The author in a letter to his fon Dr. Donald Monro, dated June 11, 1766, gave the following account of his cafe:

"Dear Donald,

"I am at prefent confined to the houfe, after "a fevere feverifh diforder. My ftate has been "fuch for fome years paft, that I have expected "to be laid afide from bufinefs, and that my "life will not be of long duration; but I fhall "little regret my difmiffion from this world, if "you are all once in an eafy way of living. "My cafe is this: In May 1762, I had the epi- "demic influenza, which affected principally "the parts in my pelvis, fo that I had a diffi- "culty and fharp pain in making water and "going to ftool. My belly has never fince been "in a regular way, paffing fometimes for feveral "days nothing but bloody mucus, and that with "confiderable tenefmus; then after fuffering fe-

"vere

" vere gripes, difcharging as much fæces as I
" would have done formerly in a week, without
" fcybala or water. Several times thefe gripes
" produced fomething very like to the iliac
" paffion ; to wit, a fevere fixed pain, three
" inches below the navel, and a little to the left
" fide, with fmart fever, vomiting &c. By
" blood-letting, and the ufe of cooling and lax-
" ative medicines and clyfters, &c. thefe firft
" violent attacks were in a great meafure got
" the better of, but left behind them an irregu-
" larity and ftoppage of ftools. About a year
" ago I was attacked with a frequent defire of
" making water, attended with heat and pain;
" and the frequent calls to thefe difcharges made
" me decline being engaged in company. Laft
" harveft I happened to obferve my urine to be
" of a very red colour; and on examining it
" found fmall knots of mucus and blood mixed
" with it, in which way it has continued ever
" fince. I found no change from diet, or any
" medicines I took, which were laxative, mild,
" mucilaginous decoctions, bark, flowers of ful-
" phur, uva urfi, &c. About a fortnight ago
" I had the addition of a fwelling and inflam-
" mation of my right *teftis*, the pain of which
" was removed by bleeding and emollient fo-

 mentations

" mentations and cataplasms, though a little of

" the swelling still continues. No external

" hæmorrhoids appear, and, so far as the finger

" can reach, there are no tubercles nor hard

" knots within the rectum.

" You know I was always subject to feverish

" disorders; and had several times the hæmoptoë

" in my early manhood, and afterwards had se-

" veral times the piles; and some bloody evacu-

" ations have been made by stool since the year

" 1762. I never had any uneasiness in my kid-

" neys, and have no right to gout or gravel either

" from my parents or from my own way of life.

" What do you think may be the disorder, and

" proper method of cure?

" Whatever may be my state, believe me that
" your welfare will ever be one of my great
" concerns.

" I am your's affectionately,

" Alex. Monro."

After this, variety of medicines were recommended to him, but all without effect; his disorder gained ground daily, with an increase of pain and uneasiness, attended with various symptoms at different times, viz. purging, fainting fits, costiveness, difficulty of making water, &c. He passed for some time blood and mucus, and thin matter matter likewife mixed with his water; and fome time before his death, the urine ufed to be mixed with air and excrement, and frequently it came away by ftool.

During this long and painful diforder he never once repined at his fate, but confcious of having acted an upright part, and of having fpent his life in the conftant exercife of his duty, he viewed death without horror, and talked of his own diffolution with the fame calmnefs and eafe as if he had been going to fleep.

On opening his body after death, there were found a preternatural adhefion of the rectum to the upper and back part of the bladder; a fungous ulcerous appearance, two fingers breadth, occupying the whole circle of the rectum, in which the difeafe probably began; and an opening, above an inch in diameter, from the rectum into the top of the bladder, which laft was otherwife found.

Of the works of our author the firft and greateft is his treatife on the anatomy of the bones; which was originally publifhed in the year 1726, for the ufe of the ftudents who attended his lectures. In this performance he has not confined himfelf to a mere defcription of the bones, bones, but has added many practical obfervations. It paffed through eight different editions during his life-time, and was tranflated into moft of the European languages; and the French edition in large folio by M. Sue is adorned with mafterly engravings of the bones.

To the later editions of his ofteology he annexed a *neurology*, or anatomy of the nerves, in which he defcribes minutely the larger branches of the particular nerves; avoiding the defcription of the very minute branches, as being apt to confound young ftudents, for whofe improvement he wrote. In this treatife he alfo mentioned moft of the prevailing opinions concerning their ftructure and ufe; and endeavoured to account for many fymptoms obferved in difeafes from their fympathy and mutual connexion. He added to this work a defcription of the receptacle of the chyle and thoracic duct.

Soon after the eftablifhment of the hofpital, a fociety was formed for collecting and publifhing Medical Effays, and they appointed Dr. Monro to be their fecretary. For the firft year the members attended the meetings, and revifed and made remarks on the papers prefented to them; but after the publication of the firft volume in 1732, they grew remifs in their attendance, fo that very foon

ſoon the whole of the collection fell upon the ſecretary.

After the publication of ſix volumes of thoſe Eſſays, the plan of the ſociety was enlarged by receiving philoſophical as well as medical papers, and it aſſumed the title of THE PHILOSOPHICAL SOCIETY. The meetings were interrupted by the rebellion in 1745, but revived again in 1752, [when our author became one of their vice-preſidents. In the two firſt volumes of *Eſſays and Obſervations Phyſical and Literary*, publiſhed by this ſociety, we find ſeveral papers written by our author, which, together with his numerous pieces inſerted in the Medical Eſſays, are reprinted in the preſent collection.

His laſt publication was his *Account of the Succeſs of Inoculation in Scotland*; which was originally written in anſwer to the delegates of the faculty of phyſicians at Paris appointed to examine into the merits of that practice. It was afterwards publiſhed at the deſire of ſome of his friends, and had a good effect in rendering this very uſeful practice more univerſal than it had formerly been in Scotland.

